# Risk, Incidence, and Mortality of Breast Cancer in Primary Sjögren’s Syndrome: A Systematic Review and Meta-Analysis

**DOI:** 10.3389/fimmu.2022.904682

**Published:** 2022-07-01

**Authors:** Jian Deng, Mengsi Liu, Ruoyi Xiao, Jin Wang, Xibei Liao, Zhen Ye, Zhen Sun

**Affiliations:** ^1^ Department of Thyroid Breast Surgery, The Second Affiliated Hospital, Hengyang Medical School, University of South China, Hengyang, China; ^2^ Center for Breast Cancer Prevention and Treatment, The Second Affiliated Hospital, Hengyang Medical School, University of South China, Hengyang, China; ^3^ Department of Clinical Medicine, Hengyang Medical School, University of South China, Hengyang, China

**Keywords:** primary Sjögren’s syndrome (pSS), breast cancer, risk factor, protective factor, mortality, incidence, meta-analysis

## Abstract

**Background:**

Primary Sjögren’s syndrome (pSS) and breast cancer are a highly prevalent autoimmune disease and malignancy, respectively, both occurring predominantly in females. Whether there is a link between these two diseases is uncertain. We conducted a systematic review and meta-analysis to investigate the risk, incidence, and mortality of breast cancer in patients with pSS.

**Methods:**

We systematically searched Embase, PubMed, and Web of Science on January 31, 2022 to identify the study that assessed risk, incidence, or mortality of breast cancer in pSS. The fixed or random-effects models were applied to pool the effect estimates based on heterogeneity measured by Cochran’s Q-test and Higgins’ I^2^.

**Results:**

Ten studies involving 725,805 participants and 64,836 pSS patients were included in our analysis. The pooled result showed that, overall, pSS was not associated with the risk (SIR=0.92, 95%CI: 0.66-1.29, *P*=0.646) and mortality (HR = 0.78, 95%CI: 0.26-2.34, *P* = 0.664) of breast cancer; however, when stratified by geographic region, we found that patients with pSS in Asian countries (SIR=1.32, 95%CI: 1.10-1.58, *P*=0.003) and Argentina (SIR=3.76, 95%CI: 1.04-9.45, *P*=0.019) had an elevated risk of breast cancer, while pSS in Europe was associated with a reduced risk (SIR=0.61, 95%CI: 0.51-0.73, *P*<0.001). The pooled result from 28,635 female pSS patients indicated that the incidence of breast cancer was 2.15 (95% CI: 1.33-3.50) per 1000 person/years.

**Conclusion:**

This study suggests that there may be geographical differences in the association between pSS and breast cancer risk; patients with pSS in European countries are associated with a lower risk of breast cancer, while Asia and Argentina are the opposite. Future research is needed to further characterize the effect of pSS on breast cancer risk and the pathophysiological mechanisms underlying this association to unravel the complex relationship between the two.

## Introduction

Primary Sjögren’s syndrome (pSS) is one of the most common systemic autoimmune diseases characterised by dryness of exocrine glands such as the lacrimal and salivary glands (1). The pSS affects 61 per 100,000 inhabitants, with a female/male ratio of approximately 10:1 (2). Previous studies have confirmed that pSS is a strong risk factor for hematological malignancies, but its association with solid tumors is still uncertain (3). A cross-sectional study showed that the most common solid cancer in pSS patients is breast cancer, which involved 0.2% of pSS patients (4).

Female breast cancer has overtaken lung cancer as the leading cause of cancer incidence worldwide by 2020 (5). Both pSS and breast cancer are common diseases in women and any possible link between the two would have a significant impact on health practice. The characteristics of breast cancer in patients with pSS were not systematically described, so we reviewed available data and conducted this meta-analysis to systematically assess breast cancer risk, incidence, and mortality in patients with pSS.

## Methods

This systematic review and meta-analysis was reported based on the Preferred Reporting Items for Systematic Reviews and Meta-Analyses guidelines (6). The protocol for this study is not registered.

### Search Strategies

Two authors systematically searched Embase, PubMed, and Web of Science databases for relevant papers published up to January 31, 2022. To minimize the risk of omitting potentially eligible papers, we combined subject terms and free words for pSS and neoplasm to develop search strategies (**Table S1**). In addition, we performed a forward search by manually checking the reference lists of eligible papers and relevant reviews to identify additional studies.

### Eligibility Criteria

Studies that met the following criteria would be considered: (a) the study population was breast cancer or pSS patients; (b) the risk, incidence, and/or mortality of breast cancer in pSS patients were the result of interest; (c) the effect size and corresponding confidence intervals (CI) of the above endpoints were provided; (d) For comparisons of breast cancer incidence and mortality in pSS vs. non-pSS populations, the study should be comparative cohort or case-control design; for assessment of breast cancer incidence in pSS, single-arm studies or cohort studies that provided breast cancer incidence in pSS cohort were considered.

Case reports/series and reports with fewer cases in studies with overlapping data would be excluded.

### Data Extraction and Quality Assessment

Two authors independently extracted the following data of eligible studies: first author, publication year, region, age, gender, source of study population, study period, study design, identification of patients with pSS and breast cancer, follow-up, endpoints, effect estimates, and confounding factors considered.

The quality of eligible studies was assessed by the Newcastle-Ottawa scale (NOS), with an NOS score >7 considered to be a high-quality study.

### Statistical Analysis

All statistical analyses were performed by Stata/MP 16.0. We checked heterogeneity of published studies by Cochran’s *Q*-test and Higgins’ *I^2^*. Random-effects models were used to pooled effect sizes to provide a more conservative risk estimate when *I^2^* > 50% or *P*< 0.1, otherwise the fixed-effects model was used. The stability of the pooled results was assessed by excluding one study at a time and then re-combining the remaining studies. Funnel plots and Begg’s and Egger’s tests were performed to check for risk of publication bias and small study effects, respectively.

## Results

**Figure 1:**
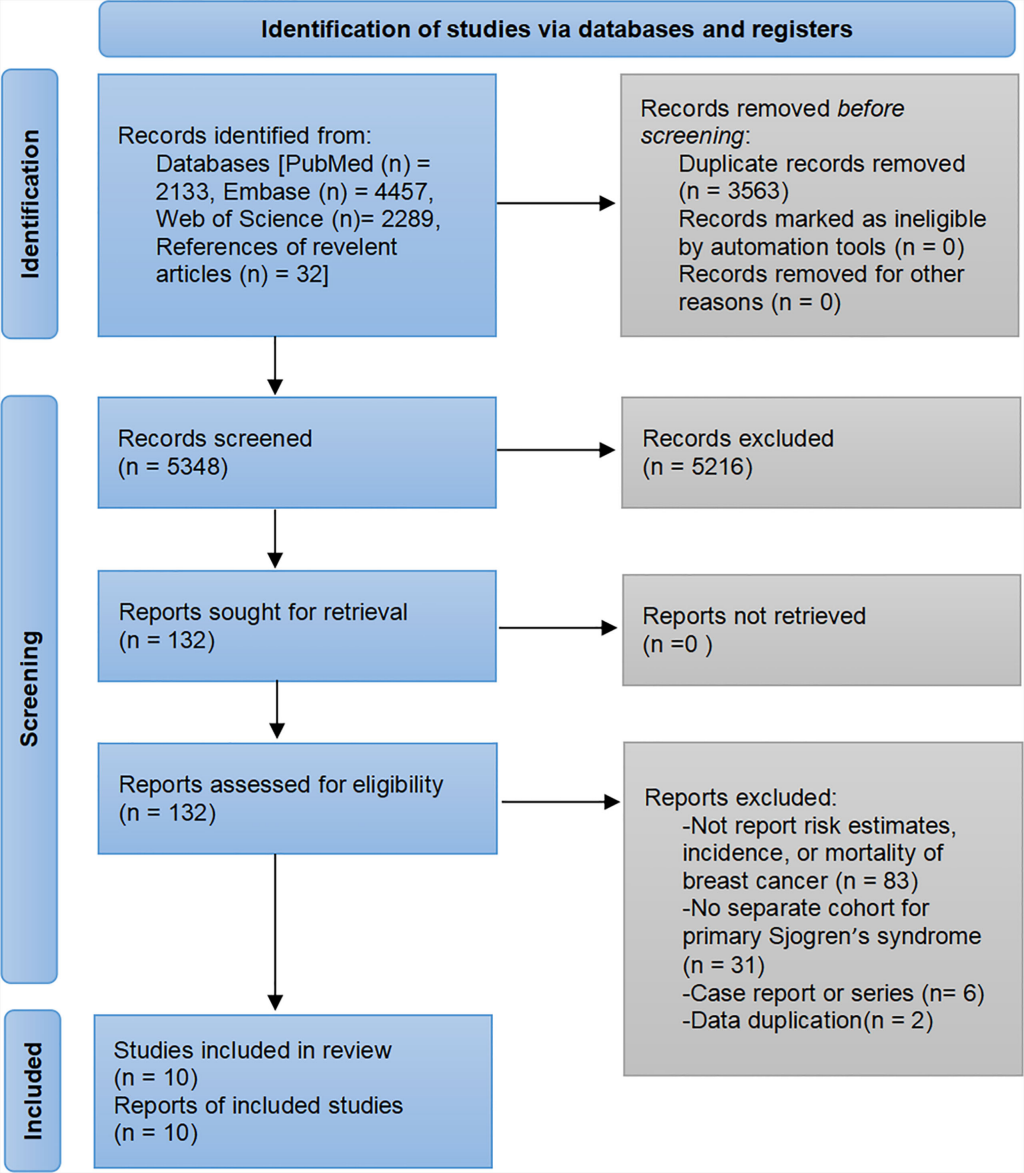
Flow diagram of the study selection process.

The pre-developed search strategy initially identified 8911 records. A total of 10 studies were ultimately included in the meta-analysis based on the inclusion and exclusion criteria (7–16). The flowchart of study selection is presented in **Figure 1**.

### Characteristics of Included Studies

The ten eligible studies involved 725,805 participants, of whom 691,387 were women and 64836 had pSS. Nine studies explored the risk of breast cancer in patients with pSS, three assessed the incidence of breast cancer, and two compared the mortality of pSS patients with other patients among breast cancer. There were four studies each in Asia and Europe, and two studies from the USA and Argentina. Seven studies were population-based cohort or case-control studies with sample sizes ranging from 1516 to 409,929; three were hospital-based cohort studies ranging from 157 to 430. The mean follow-up time ranged from 3.1 to 11.1 years. The detailed characteristics of the included studies are presented in **Table 1**.

**Table 1:** Characteristics of included studies.

Study (year)	Region	Period	Study design	Age-years	Participant source	pSS/cases	non-pSS/controls	Diagnosis of pSS	Diagnosis of breast cancer	Matched/adjusted confounding factors	Endpoints	Follow-up-years
Brito-Zerón, 2017 (7)	Spanish	2005-2016	Population-based cohort study	Mean 55.1	GEAS-SS Study Group	1300	NA	AECG	ICD-10	Age and sex	Risk (SIR)	Mean 7.6
Goulabchand, 2021 (8)	France	2011-2018	Population-based cohort study	Mean 60.0	French health insurance database	25661	252543	ICD-10 M350	ICD-10 C50	Age, sex, calendar period, socioeconomic status, annual hospitalization rate, diabetes, hypertension, cardiovascular diseases, and neuropsychiatric disorders	Risk, incidence, mortality	Median 4.0
Kang, 2020 (9)	Korea	2012-2015	Population-based cohort study	Mean 60.7	Health Insurance Review and Assessment Service database	6359	NA	ICD-10 M35.0 and RID-V139	ICD-10 C50	NA	Incidence	Mean 3.1
Theander, 2006 (10)	Sweden	1984-2002	Hospital-based cohort study	Median 56	Outpatient clinic of Malmo¨ pSS Register	286	NA	AECG	ICD-7 170	Age, sex, and calendar period	Risk (SIR)	Median 8
Brom, 2019 (11)	Argentina	2000-2017	Hospital-based cohort study	Mean 57.8	A large teaching hospital	157	NA	AECG	ICD-10	Age and sex	Risk (SIR), incidence	Mean 7.4
Wang, 2020 (12)*	Taiwan, China	1998-2013	Population-based cohort study	Mean 54.1	National Health Insurance	15636	NA	ICD-9 710.2	ICD-9	Age and sex	Risk (SIR)	Mean 5.6
Yu, 2016 (13)*	Taiwan, China	1997-2012	Population-based cohort study	Mean 53.5	National Health Insurance Research Database	11988	NA	ICD-9 710.2	ICD-9	Age and calendar period	Risk (SIR)	Mean 6.1
Aslan, 2021 (14)	Turkey	2004-2018	Hospital-based cohort study	Mean 58.6	Akdeniz University Hospital	430	NA	American College of Rheumatology 2012 criteria	ICD-10	Age and sex	Risk	Mean 7.4
Hemminki, 2012 (15)	Sweden	1964-2008	Population-based cohort study	NP	Swedish national datasets	1516	NA	ICD	ICD	Age, calendar period, region, socioeconomic status, obesity, chronic obstructive pulmonary disease, alcoholism, parity, and age at first childbirth	Risk (SIR), mortality	Mean11.1
Schairer, 2017 (16)	USA	1992–2011	Population-based case-control study	Median 76.0	SEER–Medicare database	209929	200000	ICD-9 710.2	ICD	Age, race, region, year of diagnosis/selection, months between entry and diagnosis/selection, mammogram, diabetes, dyslipidemia, autoimmune conditions	Risk	Mean5.5

pSS, primary Sjogren’s syndrome; GEAS-SS, Autoimmune Diseases Study Group-Sjogren’s syndrome; ICD, International Classification of Diseases; RID, intractable disease code; AECG, American-European Consensus Group Criteria; SEER, Surveillance, Epidemiology and End Results; NA, not applicable; NP, not reported; SIR, Standardized incidence.

*The two studies had similar sample sources and study periods; however, Wang et al. provided an assessment of breast cancer risk in all patients and Yu et al. was in women, so they were included in the overall and female subgroup analyses, respectively.

With the exception of one single-arm study assessing incidence (NOS score of 6) (9), all studies that compared breast cancer risk or mortality had NOS scores of 8 or 9, indicating that the included studies were of reasonable quality (7, 8, 10–16). Overall, these studies had appropriate selection of exposed and unexposed cohorts, good comparability, and enough follow-up for outcomes (**Table S2**).

### Breast Cancer Risk

**Figure 2:**
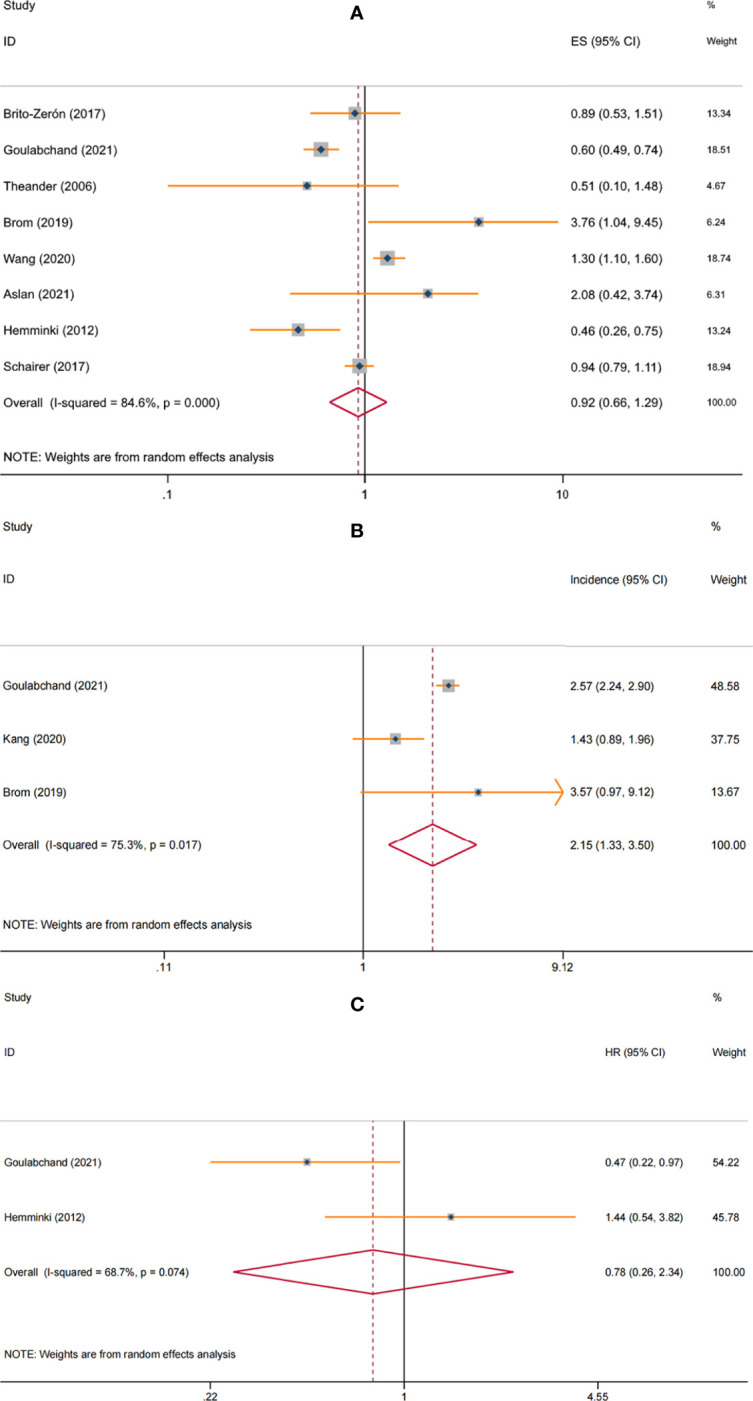
Forest plot of the associations between primary Sjögren’s syndrome and breast cancer: **(A)** risk; **(B)** incidence; **(C)** mortality.

Eight studies involving 46,489 pSS patients underwent breast cancer risk assessment (7, 8, 10–12, 14–16). Heterogeneity was significant (*I^2 =^* 84.6%, *P*<0.001), so the random-effects model was used. The overall pooled result from the random-effects model indicated that breast cancer risk was comparable between pSS and non-pSS patients (SIR=0.92, 95%CI: 0.66-1.29, *P*=0.646) (**Figure 2A**).

When restricted to the female pSS patients, breast cancer risk remained similar to the general population (SIR=0.93, 95%CI: 0.67-1.28, *P*=0.656). In the non-specific gender subgroup, breast cancer risk was elevated in pSS patients (SIR=1.29, 95% CI: 1.08-1.55, *P*=0.006), which may be due to common risk factors, with female involvement predominating in both diseases. Of note, the correlation between pSS and breast cancer risk appeared to be influenced by geographic regions. When stratified analysis was performed according to study location, we found a significantly lower risk of breast cancer among pSS patients in European countries (SIR=0.61, 95%CI: 0.51-0.73, *P*<0.001), while significantly increased risks in Asian countries (SIR=1.32, 95%CI: 1.10-1.58, *P*=0.003) and Argentina (SIR=3.76, 95%CI: 1.04-9.45, *P*=0.019); no correlation between pSS and breast cancer risk was observed in the USA (SIR=0.94, 95%CI: 0.79–1.11, *P*=0.476). For studies that adjusted only for age and sex, the pooled result was not significant (SIR=1.39, 95%CI: 0.91-2.13, P=0.129), while the overall result from studies that considered confounders beyond these two (such as study period, socioeconomic status, annual hospitalization rate, or chronic disease) showed a lower risk of breast cancer in patients with pSS (SIR=0.66, 95% CI: 0.46-0.96, *P*=0.028). Furthermore, hospital-based studies observe a higher risk of pSS than population-based studies (SIR: 1.68, 95% CI: 0.57-5.01 vs. 0.81, 95% CI: 0.57-1.15, respectively). No significant differences were observed at different ages and follow-up (**Table 2**).

**Table 2:** Stratified analysis of the breast cancer risk in patients with primary Sjögren’s syndrome.

Subgroups	No. of Studies	SIR (95%CI)	*P _Z_*	Heterogeneity (*I* ^2^, *P_H_*)	Effects model	Meta-regression (*P*_interaction_)^*^
*Gender*						0.355
Female	7	0.93 (0.67-1.28)	0.656	83.1%, <0.001	Random	
Unspecific	3	1.29 (1.08-1.55)	**0.006**	21.8%, 0.278	Fixed	
*Matched/adjusted confounder*						0.696
Age, sex	4	1.39 (0.91-2.13)	0.129	52.2%, 0.099	Random	
More than age and sex	4	0.66 (0.46-0.96)	**0.028**	79.8%, 0.002	Random	
*Median/mean age*						0.289
<60 years	5	1.29 (0.84-1.98)	0.239	50.5%, 0.088	Random	
≥60 years	2	0.75 (0.49-1.17)	0.208	90.8%, <0.001	Random	
*Study design*						0.246
Population-based	5	0.81 (0.57-1.15)	0.236	88.9%, <0.001	Random	
Hospital-based	3	1.68 (0.57-5.01)	0.348	61.1%, 0.076	Random	
*Region*						0.078
Europe	4	0.61 (0.51-0.73)	**<0.001**	5.7%, 0.364	Fixed	
Asia	2	1.32 (1.10-1.58)	**0.003**	0.0%, 0.407	Fixed	
Argentina	1	3.76 (1.04–9.45)	**0.019**	NA	NA	
United States	1	0.94 (0.79–1.11)	0.476	NA	NA	
*Followed-up*						0.429
<6 years	3	0.90 (0.60-1.37)	0.633	93.3%, <0.001	Random	
≥6 years	5	1.03 (0.51-2.11)	0.927	73.8%, 0.004	Random	

Bold indicates P values with statistical significance (<.05).

SIR, Standardized incidence ratios; NA, not applicable

*Multivariate meta-regression analysis for all the variables listed in this table.

The multivariate meta-regression analysis of the above subgroups indicated that only the geographical area was at borderline significance (*P*_interaction_=0.078), suggesting that it may be the source of heterogeneity (**Table 2**).

### Breast Cancer Incidence

Three studies involving 28,635 female pSS patients assessed breast cancer incidence (8, 9, 11). The meta-analysis with the random-effects model showed that the incidence of breast cancer was 2.15 (95% CI: 1.33-3.50) per 1000 person/years in patients with pSS, with significant heterogeneity (*I^2^* = 75.3%, *P* = 0.017) (**Figure 2B**). Subgroup analyses were not performed due to the relatively small amount of data available.

### Breast Cancer Mortality

Two studies involving 3014 female patients with breast cancer assessed the effect of pSS on mortality (8, 15). The pooled result of random-effects model showed no increased mortality among female breast cancer patients with pSS vs. non-pSS (HR = 0.78, 95%CI: 0.26-2.34, *P* = 0.664), with significant heterogeneity (*I^2^* = 68.7%, *P* = 0.074) (**Figure 2C**). However, the included studies did not specify whether the mortality was cancer-specific or all-cause. In addition, no studies assessed the impact of pSS on breast cancer prognosis.

### Sensitivity Analysis and Publication Bias

Due to the relatively small number of eligible studies in the incidence and mortality endpoints, we only performed sensitivity analysis for the risk analysis and examined small study effects based on the risk endpoint. By excluding one study at a time and then re-combining the remaining studies, we found that individual studies had little effect on the pooled results indicating that the current result was relatively stable (**Figure S1**). The *P* value of Begg’s and Egger’s tests were 1.000 and 0.922, respectively, indicating that no small study effects existed. Funnel plots to assess publication bias were not performed because none of these comparisons had at least ten studies.

## Discussion

### Principal Findings

To our knowledge, this is the first study to focus on the breast cancer risk, incidence, and mortality in patients with pSS. We found that, overall, the risk of breast cancer in pSS patients was similar to that of the general population, but there were clear geographical differences; pSS patients in European countries had a significantly lower risk of breast cancer, while the risks in Asia and Argentina were significantly increased. The incidence of breast cancer in women with pSS ranged from 1.43 to 3.57, with a pooled incidence of 2.15 (95%CI: 1.33-3.50) per 1000 person/years. Patients with pSS did not have an increased mortality rate in female breast cancer.

### Comparison With Other Reviews

A meta-analysis published in 2014 showed no association between pSS and breast cancer risk (SIR = 0.67, 95% CI: 0.27-1.06) (17). Compared to their study, our meta-analysis included many newer and larger investigations and thus had significantly greater number of participants. Although the findings in our study regarding overall breast cancer risk were consistent with theirs, we found an effect of geographic regions on this risk. Other autoimmune rheumatic diseases, including rheumatoid arthritis, systemic lupus erythematosus, and psoriasis, were also found not to be associated with breast cancer risk (18–20). Interestingly, there were differences between patients with rheumatoid arthritis by ethnicity; the risk of breast cancer was reduced in Caucasian patients with rheumatoid arthritis but increased in non-Caucasians, which appears to be similar to the geographic differences we observed (18). Unfortunately, most studies did not provide the exact ethnic composition of pSS participants, so we cannot stratify the analysis by ethnicity again. Genetic polymorphism may play a role in the correlation between pSS and breast cancer; future Mendelian randomization studies are warranted.

### Potential Interpretations of the Results

There was substantial heterogeneity in most of the pooled analyses (*I^2^* > 50%), but interestingly, the significant heterogeneity disappeared in the subgroups analysis according to geographic region, suggesting a relatively consistent trend within this stratification. Multivariate meta-regression analyses further determined geographical region may be the source of heterogeneity. All four studies from Europe showed a trend towards a lower risk of breast cancer in patients with pSS (7, 8, 10, 15). The two studies from Asia were from China and Turkey (12, 14); as most of Turkey’s territory is located in Asia, we categorized it as an Asian country. Thus, the relatively small amount of data from Asia limits the ability to draw definite conclusions. This geographical variation may be due to differences in genetics, environment, and lifestyle at the population level.

A noteworthy finding was that studies adjusting for confounders more than age and gender overall showed a lower risk of breast cancer in pSS, with additional factors considered including study period, socioeconomic status, annual hospitalization rate, chronic disease, race, area, alcoholism, parity, and/or age at first childbirth; these studies were mainly from Europe (8, 10, 15, 16). In contrast, the studies from the Asian and Argentine only adjusted for age and gender. But the difference between European and non-European populations cannot be fully attributed to the adjustment for additional confounders, because a European study that adjusted only for age and gender also found the same trend in breast cancer risk for pSS patients as other European studies (7). The meta-regression analysis also did not support differences in confounders to explain heterogeneity (*P*_interaction_=0.696). In fact, there are many other confounders associated with breast cancer, such as weight, smoking, age at marriage, and family history, that have not been adjusted for in all published studies.

In addition, differences in medical practice and policy between countries may be an important interference factor, for example, different diagnostic criteria and treatment management for pSS. Most of the current studies used read diagnostic codes, such as the International Classification of Diseases code, to identify patients with pSS and cancer, and pSS was diagnosed according to the American-European Consensus Group (AECG) criteria. Although differences in medical practice between countries are difficult to quantify, an interesting finding is that studies based on a single reference hospital tended to report a higher relative risk of breast cancer compared to population-based studies (SIR: 1.68 vs. 0.81, respectively); studies from Turkey and Argentina with participants recruited from tertiary care centers both showed an effect size of more than 2 for the correlation between pSS and breast cancer risk, which suggests that the severity and activity of pSS may affect breast cancer risk (21). However, this cannot explain the positive association between pSS and breast cancer risk in non-European populations, since the Taiwan study based on the health insurance database also showed a significantly higher risk of breast cancer in pSS patients and the regression analysis did not support that the cohort source led to significant heterogeneity (*P*_interaction_=0.246). Moreover, an important hypothesis is that there were effects of therapeutic agents for pSS including glucocorticoids, non-steroidal anti-inflammatory drug (NSAIDs), methotrexate, biologics, and disease modifying anti-rheumatic drugs on breast cancer risk. It has been shown that NSAIDs appear to have a protective effect on breast cancer, while immunosuppressive therapy may increase the risk of developing opportunistic infections and cancer (22, 23). Thus, medication preferences for pSS in different regions may be a key factor influencing breast cancer risk. In addition, although subgroup analysis by mean/median follow-up time showed that follow-up time did not affect the risk of breast cancer in pSS, future studies should conduct stratified analyses based on the condition duration to explore the long-term effects and to exclude reverse causality.

### Implications

Overall, due to the underrepresentation of populations outside Europe and the heterogeneity of study designs in different regions, the association between pSS and breast cancer risk may be coincidental, so the pooled results need to be interpreted with caution. However, given the alarmingly increasing incidence of breast cancer and the large population of patients with pSS, additional attention should be paid to such risk in regions where pSS has been found to be a possible risk factor for breast cancer. Future studies can further explore the correlation between the relevant clinical, histological, and laboratory features of pSS patients and breast cancer risk, which may help clinicians accurately identify those at high risk of developing breast cancer; periodic surveillance and rigorous screening of this population can facilitate early diagnosis and appropriate therapeutic management of the malignancy, thereby improving survival rates. In addition, the inverse correlation between pSS and breast cancer risk found in European studies suggests that there may be pathophysiological interactions between pSS and breast cancer, and further basic and translational research on the underlying mechanisms may provide new targets for breast cancer prevention and treatment.

### Amendments to the Pre-Planned Protocol and Limitations of This Study

This study followed a pre-planned but unpublished protocol that can be accessible by contacting the corresponding author. The primary study objective was to examine the risk, incidence, and mortality of breast cancer in patients with pSS; the secondary objective was to characterize the association between pSS and breast cancer, so stratified analyses according to age, gender, region, study design, and follow-up were pre-planned. Analyses that were not performed after seeing the preliminary results were sensitivity analyses for incidence and mortality endpoints and funnel plots for publication bias; the exclusion of these analyses were due to the concern about lack of statistical power resulting from the relatively small number of eligible studies. Added analyses included stratified analyses based on confounding factors and multivariate meta-regression analyses; these analyses were performed to assess the effects of confounding factors on outcomes and to identify causes of heterogeneity. In addition, as discussed above, we also described as comprehensively as possible the effects of various confounding factors on published studies and carefully interpreted the findings to provide a balanced and critical perspective on the subject.

The main limitation of this study is the lack of detail on the association between pSS and breast cancer. Because there are no studies specifically focusing on breast cancer among patients with pSS, almost all studies on this topic examined the risk of multiple malignancies in patients with pSS or the risk of breast cancer in multiple autoimmune diseases; of them, breast cancer or pSS is only a subanalysis. Therefore, the characteristics of breast cancer risk in patients with pSS are unclear; factors affecting the association between pSS and breast cancer risk, including the effects of treatment, severity/activity, and duration of pSS, remain to be elucidated. Many other confounding factors such as weight, smoking, family history, alcohol consumption, age at marriage, and gynecological history may bias our results. Representation of the population outside Europe is relatively insufficient. In addition, data on the prognosis and mortality of breast cancer patients with concurrent pSS are scarce. This study protocol is not pre-registered, but we have completed the pre-planned analyses as far as possible when sufficient data is available, so the results are unlikely to be biased due to methodology. Finally, although Begg’s and Egger’s tests indicated no small study effect, potential risk of publication bias may still exist.

## Conclusion

This study suggests that there may be geographical differences in the association between pSS and breast cancer risk; patients with pSS in European countries are associated with a lower risk of breast cancer, while Asia is the opposite. Future studies are needed to explore the characteristics of the association and underlying pathophysiologic mechanisms between pSS and breast cancer, particularly the impacts of disease severity, activity, and management of pSS, which may provide new insights into breast cancer prevention and treatment.

## Data Availability Statement

The original contributions presented in the study are included in the article/**Supplementary Material**. Further inquiries can be directed to the corresponding authors.

## Author Contributions

Conception and design: ZS, ML. Acquisition of data: JW, JD, Statistical analysis: ZY, XL. Drafting of manuscript: JD, ML, ZS, RX. Critical revision of the manuscript: ZS. All authors contributed to the article and approved the submitted version.

## Conflict of Interest

The authors declare that the research was conducted in the absence of any commercial or financial relationships that could be construed as a potential conflict of interest.

## Publisher’s Note

All claims expressed in this article are solely those of the authors and do not necessarily represent those of their affiliated organizations, or those of the publisher, the editors and the reviewers. Any product that may be evaluated in this article, or claim that may be made by its manufacturer, is not guaranteed or endorsed by the publisher.
