# Supplementary material for: Risk, Incidence, and Mortality of Breast Cancer in Primary Sjögren’s Syndrome: A Systematic Review and Meta-Analysis
Source: Front Immunol. 2022 Jul 1;13:904682. doi: 10.3389/fimmu.2022.904682 (PMC9283727; doi:10.3389/fimmu.2022.904682)
Supplement: Supplementary file 1 [file DataSheet_1.docx]

| **Table S1.** Specific search strategy for each database | |
| --- | --- |
| **PubMed** | ("Neoplasms"[Mesh] OR Neoplasia[Title/Abstract] OR Neoplasias[Title/Abstract] OR Neoplasm[Title/Abstract] OR Tumors[Title/Abstract] OR Tumor[Title/Abstract] OR Cancer[Title/Abstract] OR Cancers[Title/Abstract] OR Malignancy[Title/Abstract] OR Malignancies[Title/Abstract] OR Malignant Neoplasms[Title/Abstract] OR Malignant Neoplasm[Title/Abstract] OR Neoplasm, Malignant[Title/Abstract] OR Neoplasms, Malignant[Title/Abstract] OR Benign Neoplasms[Title/Abstract] OR Neoplasms, Benign[Title/Abstract] OR Benign Neoplasm[Title/Abstract] OR Neoplasm, Benign[Title/Abstract]) AND ("Sjogren's Syndrome"[Mesh] OR Sjogrens Syndrome[Title/Abstract] OR Syndrome, Sjogren's[Title/Abstract] OR Sjogren Syndrome[Title/Abstract] OR Sicca Syndrome[Title/Abstract] OR Syndrome, Sicca[Title/Abstract]) |
| **Embase** | ('malignant neoplasm'/exp OR 'cancer':ab,ti OR 'cancers':ab,ti OR 'malignant neoplasia':ab,ti OR 'malignant neoplastic disease':ab,ti OR 'malignant tumor':ab,ti OR 'malignant tumour':ab,ti OR 'neoplasia, malignant':ab,ti OR 'tumor, malignant':ab,ti OR 'tumour, malignant') AND ('Sjoegren syndrome'/exp or 'dacryosialoadenopathia atrophicans':ab,ti OR 'dyssecretosis, mucoserous':ab,ti OR 'gougerot houwer sjoegren syndrome':ab,ti OR 'gougerot mulock houwer sjoegren syndrome':ab,ti OR 'gougerot sjoegren disease':ab,ti OR 'Gougerot Sjoegren syndrome':ab,ti OR 'gougerot sjogren disease':ab,ti OR 'Gougerot Sjogren syndrome':ab,ti OR 'Gougerot-Sjogren syndrome':ab,ti OR 'mikulicz gougerot sjoegren syndrome':ab,ti OR 'mikulicz radecki syndrome':ab,ti OR 'mucoserous dyssecretosis':ab,ti OR 'mukilicz radecki syndrome':ab,ti OR 'oculobuccopharyngeal dryness':ab,ti OR 'rheumatic sialosis':ab,ti OR 'sialosis, rheumatic':ab,ti OR 'sicca syndrome':ab,ti OR 'sjoegren disease':ab,ti OR 'sjogren disease':ab,ti OR 'sjogren syndrome':ab,ti OR 'sjogrens syndrome':ab,ti)4457 |
| **Web of Science** | TS= ((Neoplasms OR Neoplasia OR Neoplasias OR Neoplasm OR Tumors OR Tumor OR Cancer OR        Cancers OR Malignancy OR Malignancies OR Malignant Neoplasms OR Malignant Neoplasm OR Neoplasm, Malignant OR Neoplasms, Malignant OR Benign Neoplasms OR Neoplasms, Benign OR Benign Neoplasms OR Neoplasms, Benign) AND (Sjogren's Syndrome OR Sjogrens Syndrome OR Syndrome, Sjogren's OR Sjogren Syndrome OR Sicca Syndrome OR Syndrome, Sicca OR dacryosialoadenopathia atrophican OR dyssecretosis, mucoserous OR gougerot houwer sjoegren syndrome)) |

| **Table S2.** The quality assessment of included studies | | | | | | | | | |
| --- | --- | --- | --- | --- | --- | --- | --- | --- | --- |
| **Study (cohort)** | **Representativeness of exposed cohort** | **Selection of non-exposed cohort** | **Ascertainment of exposure** | **Outcome not present before study** | **Comparability** | **Assessment of outcome** | **Follow-up long enough*** | **Adequacy of follow up** | **Quality score** |
| Brito-Zerón, 2017 | ★ | ★ | ★ | ★ | ★☆ | ★ | ★ | ★ | 8 |
| Goulabchand, 2021 | ★ | ★ | ★ | ★ | ★★ | ★ | ★ | ★ | 9 |
| Kang, 2020 | ★ | ☆ | ★ | ★ | ☆☆ | ★ | ★ | ★ | 6 |
| Theander, 2006 | ★ | ★ | ★ | ★ | ★★ | ★ | ★ | ★ | 9 |
| Brom, 2019 | ★ | ★ | ★ | ★ | ★☆ | ★ | ★ | ★ | 8 |
| Wang, 2020 | ★ | ★ | ★ | ★ | ★☆ | ★ | ★ | ★ | 8 |
| Aslan, 2021 | ★ | ★ | ★ | ★ | ★☆ | ★ | ★ | ★ | 8 |
| Yu,2016 | ★ | ★ | ★ | ★ | ★★ | ★ | ★ | ★ | 9 |
| Hemminki, 2012 | ★ | ★ | ★ | ★ | ★★ | ★ | ★ | ★ | 9 |
| **Study (case-control)** | **Case definition** | **Representativeness of the cases** | **Selection of Controls** | **Definition of Controls** | **Comparability** | **Ascertainment of exposure** | **Same method of ascertainment** | **Non-Response rate** | **Quality score** |
| Schairer, 2017 | ★ | ★ | ★ | ★ | ★★ | ★ | ★ | ★ | 9 |
| * Median/mean follow-up of more than 3 years or maximum follow-up of more than 5 years was considered enough. | | | | | | | | | |


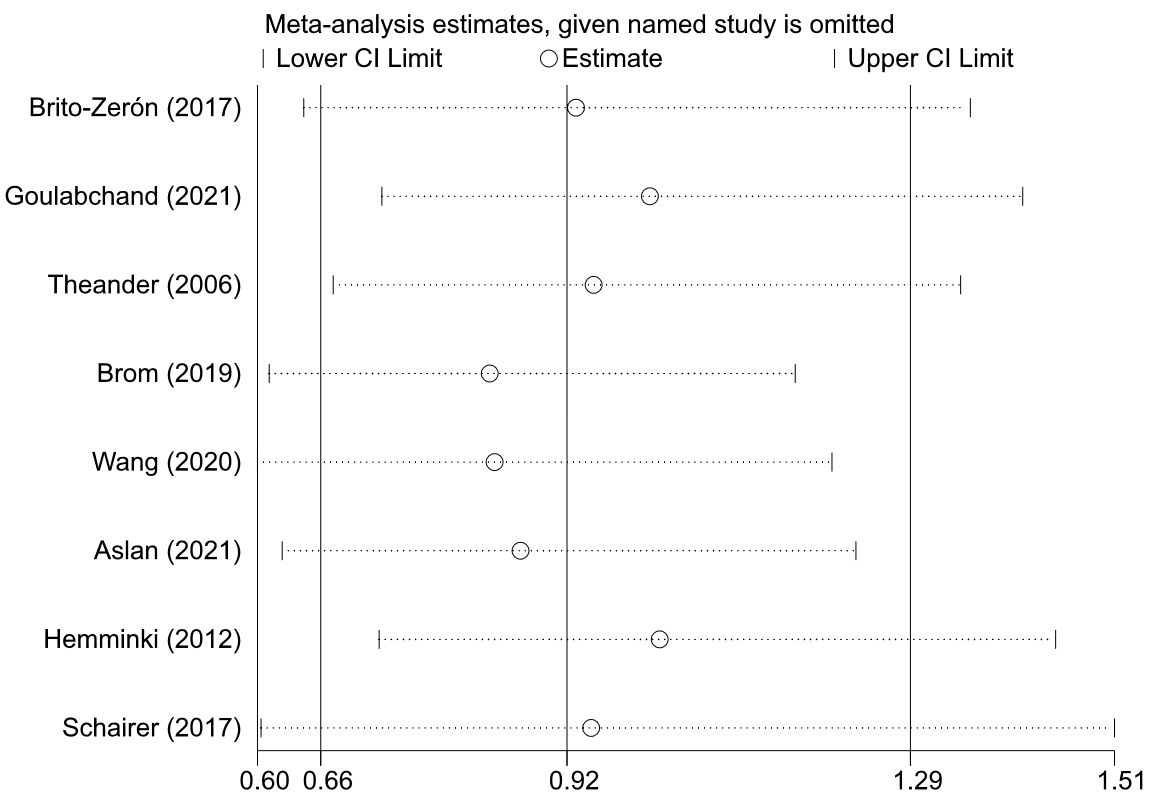


**Figure S1.** The effects of the individual studies on the overall pooled risk.
